# Meeting Review of the Cryospheric Ecosystems Conference, 1-4 September 2025, Poznań, Poland

**DOI:** 10.1242/bio.062385

**Published:** 2026-05-11

**Authors:** Kinga Skoczeń, Zofia Kujawa, Magdalena Strawa, Filip Marcinkowski, Selina Tinkhauser, Mikołaj Jastrzębski, Róża Ulikowska

**Affiliations:** ^1^ Department of Animal Taxonomy and Ecology, Adam Mickiewicz University in Poznań, 61-614 Poznań, Poland; ^2^ UiT The Arctic University of Norway, 9037 Tromso, Norway

**Keywords:** Cryosphere, Glacial ecosystems, Biodiversity, Biogeochemistry, Snow and ice algae, Climate change impacts

## Abstract

The Cryospheric Ecosystems Conference, held at Adam Mickiewicz University in Poznań from September 1 to 4, 2025, brought together 80 scientists, researchers, and students from 15 countries. The conference was held as part of the International Year of Glaciers’ Preservation 2025, providing the ideal context and platform for sharing the results and discussions on recent discoveries, emerging threats, the latest techniques, and future projects. The conference highlighted the importance of cryospheric environments, such as ice sheets, glaciers and snow, which host unique biodiversity. The event covered the cascading effects of global warming on glacier retreat and biodiversity, the role of phototrophs in snow and ice, the biogeochemical cycling of fundamental elements such as carbon, nitrogen and phosphorus, and pollutants in cryospheric environments. Here we present the key outcomes and discussions of the conference, emphasizing the importance of cryospheric ecosystems monitoring and protection.

## Introduction

Snow, glaciers, sea ice, and permafrost constitute the primary components of the cryosphere, a critical subsystem of the earth that plays a fundamental role in regulating global climate and supporting ecological balance throughout the Holocene (Barry, 2002; IPCC, 2023). These components significantly influence planetary albedo, contribute to sea-level regulation, and serve as major reservoirs of freshwater. Additionally, the cryosphere shapes adjacent ecosystems and several ecosystem functions such as nutrient recycling, productivity, and supporting a myriad of habitats for organisms (Ficetola et al., 2021; Stibal et al., 2020; Crosta et al., 2025). Glaciers and snow environments host diverse communities of bacteria and algae, enhancing and maintaining higher trophic level consumers, such as invertebrates (Ono et al., 2021; Zawierucha et al., 2022; Millar et al., 2024). Moreover, the last glaciers and snowfields in warm regions are essential habitats for psychrophilic organisms (cold-adapted specialist species), which increasingly rely on these environments as refugia under ongoing climate warming (Valle et al., 2025; Zawierucha et al., 2021; Shain et al., 2024). It is also a key domain in polar and high-mountain regions where various contaminants (organic and inorganic) can accumulate, persist, and finally be released (Buda et al., 2024; Rozwalak et al., 2022; Makowska-Zawierucha et al., 2022; Muir et al., 2025). Ice acts as a witness to hundreds of thousands of years of world history, volcanic eruptions, shifts in atmospheric composition, and landscape changes. As the Holocene ends and the Anthropocene begins, the scientific community needs to gather interdisciplinary knowledge of atmosphere–cryosphere–biodiversity and pollutant interactions in order to better understand the fate of cryospheric ecosystems. The conference directly addressed this need by fostering a consensus on the prioritization of rapid environmental shifts of the cryosphere. By providing the first platform for interdisciplinary collaboration and knowledge sharing from international researchers exclusively about cryospheric ecosystems, the conference made an important step forward in addressing the complex interconnections between the components of the cryosphere and life on earth. While the final goal of fully understanding the cryosphere and how it will change in the future because of global warming remains a long-term challenge, the outcomes of the conference represent a starting point toward achieving that vision and anticipating the impact of cryosphere loss on unique parts of biodiversity.

**Figure:**
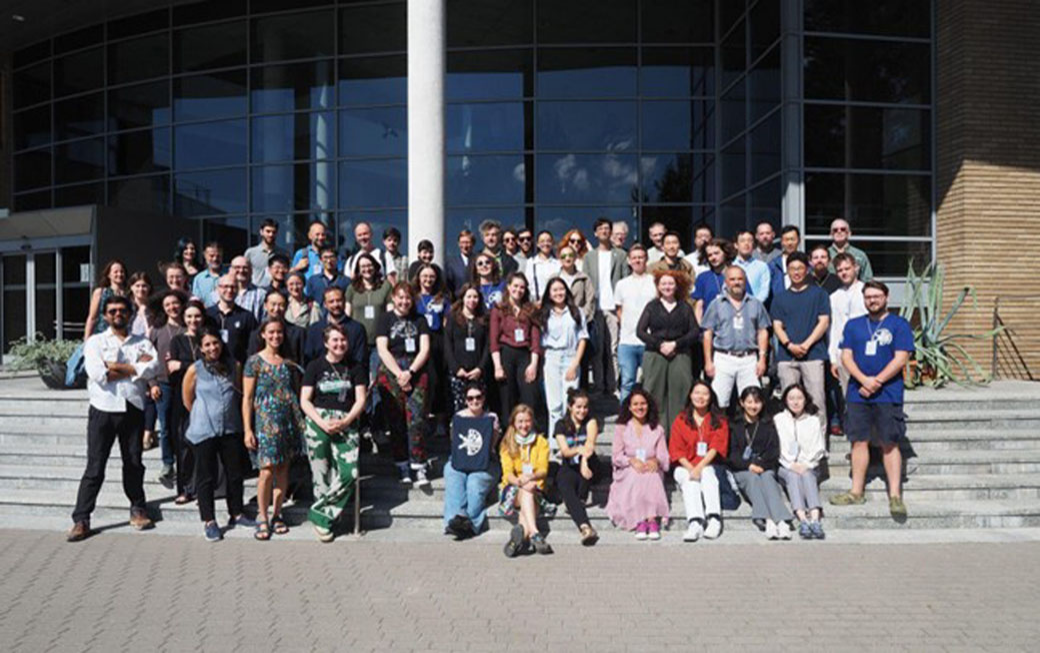
A group photograph of the in-person attendees of the Cryospheric Ecosystems Conference 2025, held at the Adam Mickiewicz University in Poznań, Poland.

**Figure:**
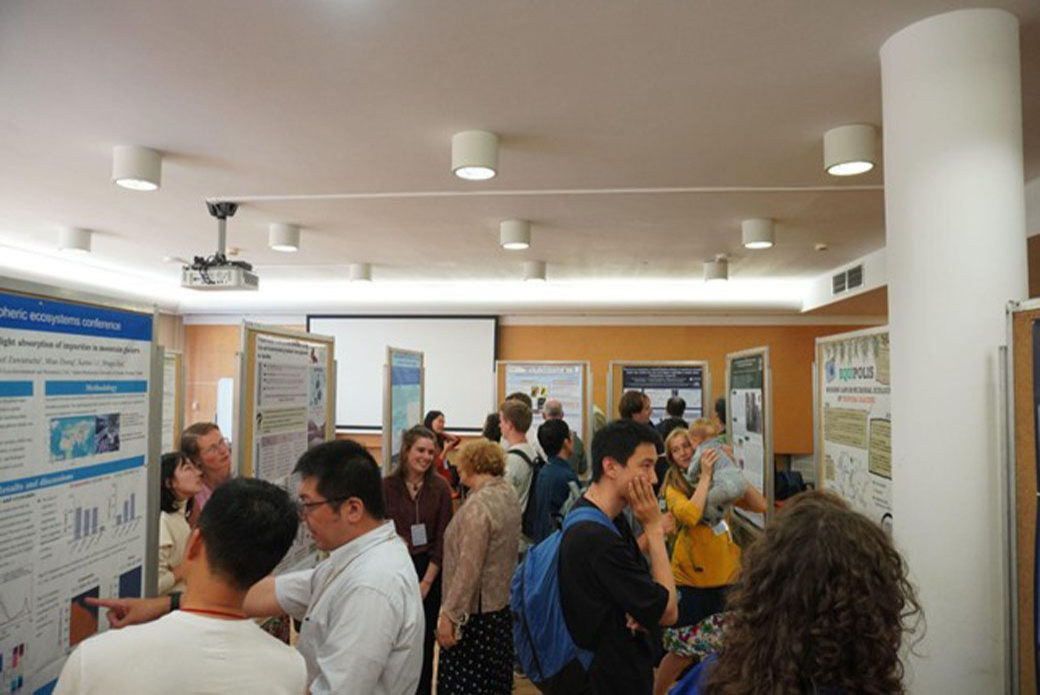
Attendees at the poster session.

## Key themes of the meeting

Topics presented during the conference covered the results of studies on biodiversity, ecology, biogeochemistry and pollution of glaciers and snow, the impact of glaciers on the adjacent systems (forefields, streams, seas), and cryosphere–atmosphere interaction. The oral presentations were organised into seven thematic sessions: (1) impact of glacier retreat on biodiversity and ecosystems; (2) phototrophs on snow, glaciers and in adjacent ecosystems; (3) carbon, nitrogen, phosphorus – on cryospheric biogeochemistry; (4) from ice cores to glacial streams: microbial diversity and dynamics in the cryosphere; (5) animals in cryospheric environments; (6) physical changes in cryospheric environments; and, (7) past, present and future of contaminants in the cryosphere.

Each session featured 15-20 minute talks by both senior and early-career researchers, followed by a short discussion.

Most of the speakers presented new data on snow algae, phototrophs on ice, microbial assemblages in cryoconite, and invertebrate communities on glaciers and glacial forefields. The focus extended to how these organisms respond to rapid environmental change – for example, melt-season shortening, habitat loss, and shifts in nutrient availability. Another major theme concerned the cycling of carbon, nitrogen, phosphorus and other elements in glacial and snow environments, and how melt, run-off and thaw link these habitats to downstream ecosystems (streams, lakes, seas). Presentations addressed the export of nutrients from ice to proglacial systems, the remobilisation of contaminants such as heavy metals and artificial radionuclides, and contaminants of emerging Arctic concern (CEACs).

A recurrent concern was the establishment of sustained, long-term monitoring programmes capable of capturing the pace and variability of change across spatial and temporal scales. Participants emphasised that meaningful synthesis across studies requires methodological standardisation, including the development of harmonised sampling protocols, consistent analytical procedures, and compatible data formats. The community gathered at the conference recognised that without such coordination, comparisons among sites and years remain limited.

Equally important was the recognition that progress in understanding cryospheric ecosystems demands a truly interdisciplinary approach. Speakers underscored that the complexity of cryospheric processes – encompassing physical, chemical, biological and climatic dimensions – cannot be fully understood within disciplinary boundaries. Integrating expertise from glaciology, ecology, microbiology, biogeochemistry, remote sensing and climate modelling is essential to link microscale biological activity with macroscale environmental dynamics. In this context, participants called for stronger collaboration across disciplines and institutions to build integrated monitoring networks and harmonised long-term series, ensuring that cryospheric ecosystem research evolves as a cohesive, transdisciplinary field capable of informing both scientific understanding and policy action.

## Speaker highlights

Four keynote speakers were invited to the opening of specific thematic sessions.

Professor Gentile Francesco Ficetola (Department of Environmental Science and Policy, University of Milan, Italy), addressed biodiversity and macroecological patterns in glacier-retreat forefields, applying eDNA of all the major taxonomic groups: bacteria, fungi, plants, protists, and soil animals to illustrate long-term ecological legacies of deglaciation. Scientists observed that all the environmental properties quickly changed with time as glaciers retreated, and that temperature modulates the accumulation of soil nutrients. Diversity of all organisms changes after deglaciation, with different patterns in the first decades microorganisms colonise the environment most rapidly, where macroorganisms took longer. Plants seem to have a key role in environmental development, showing positive links to all other organisms.

Professor Steven K. Schmidt (Ecology and Evolutionary Biology Department, University of Colorado, Boulder, CO, USA) delivered a plenary on microbial life in high-altitude and polar ice environments. This research helped answer the question of what factors: humidity, UV-radiation or nutrients, could limit phototrophic microbes and mosses in extreme ecosystems. The study shows, unexpectedly, that nutrient limitation is more important than the extreme climate in controlling microbial activity in these ecosystems, and phosphorus is the most limiting factor.

Professor Nozomu Takeuchi (Department of Earth Sciences, Graduate School of Science, Chiba University, Japan) delivered a presentation focused on variations in phototrophs' community in cryoconite holes. The audience learned that hole dimensions directly influence the microbial community and its productivity. For instance, the intensity and spectral properties of solar radiation reaching the hole's bottom, critical for phototrophs, are highly dependent on hole depth. Depth also impacts the stability and persistence of holes, which in turn significantly affects the cycling of organic matter and nutrients, thereby influencing microbial community composition and productivity. However, the factors governing their spatial variability and dimensions on glaciers are still not fully understood. Professor Takeuchi observed that phototrophs differ biogeographically and proposed the idea of creating an artificial cryoconite hole as a possible geoengineering method to mediate biological darkening and glacier melt.

Professor Daniel Shain (Biology Department, Rutgers, The State University of New Jersey, USA) presented results on the studies of ice worms on glaciers and bioenergetics of psychrophiles. A central component of ice-inhabiting life appears to be energetic, namely an ability to maintain high ATP levels that drive reactions and biological processes forward. To this end, ice worms have acquired a fragment of environmental DNA by lateral gene transfer that enhances ATP production capacity.

The final keynote speaker, Dr Bartłomiej Luks (Institute of Geophysics, Polish Academy of Sciences, Poland) underscored the critical yet often overlooked role of Arctic snow as an active interface linking the atmosphere, cryosphere and biosphere. He also highlighted the need for a true interdisciplinary approach in polar and cryospheric science and invited conference participants to collaborate across disciplines to better understand and monitor the rapidly changing Arctic environment.

## Networking opportunities

The ice-breaker session provided an opportunity for lively discussions and networking among the attendees. Many undergraduate and experienced scientists had the opportunity to meet each other for the first time. Oral and poster sessions, and coffee breaks, provided excellent opportunities to establish contacts with representatives of the scientific community engaged in cryosphere research. The food provided during the coffee breaks and lunch were vegetarian to reduce the carbon footprint. To further offset the carbon footprint, no single-use plastics were used throughout the conference and public transport tickets were provided for participants. Ice-breakers and coffee breaks were important for the younger members of the community working on cryospheric ecosystems. Throughout these informal meetings, participants exchanged ideas and shared experiences related to their ongoing projects. These discussions promoted an inspiring atmosphere, allowing participants to gain new perspectives and insights into different methodologies and research approaches. The exchange of knowledge and ideas contributed greatly to broadening scientific understanding and building a foundation for potential joint projects. Undergraduate students got internship offers, and the possibility to be involved in the studies on both glacial and proglacial ecosystems. As part of the conference's official closing ceremony, a gala dinner was organised, which served as a fitting finale to the networking activities and was accompanied by a Fryderyk Chopin music concert.

## Poster session

The poster session, which was held on Wednesday, 3 September 2025, contained 22 presentations on a range of topics, including those related to biological sciences and earth sciences. Participants introduced the results of their research, which were conducted on glaciers, glacierets, snow patches and related ecosystems from polar regions to tropical mountains. Most of the posters presented new data on the taxonomy, ecology and physiology of algae (primary producers) and invertebrates (top consumers). Posters on abiotic environments focused on contaminants such as persistent organic pollutants and dark, albedo-reducing impurities on glaciers and their chemical content.

The poster session also played an important role in supporting early-career researchers, providing an opportunity to discuss their results and methods with more experienced colleagues, and encouraging the development of international collaborations in a pleasant atmosphere. The active discussions, full of passion and new ideas, extended the poster sessions by almost two hours.

## Conclusions

The Cryospheric Ecosystems Conference emphasised the cryosphere's crucial role in regulating global climate and biodiversity. Discussions highlighted that glaciers, snow, permafrost, and sea ice are not just physical entities but dynamic ecosystems that host diverse and unique microbial communities, fauna, and flora adapted to extreme conditions. Across plenary talks and thematic sessions, new findings demonstrated complex connections between biological diversity, biogeochemical cycles, and pollutants within glacial biome. The conference underlined the importance of interdisciplinary and international collaboration, open data sharing, and predictive modelling in advancing the understanding of cryospheric change. Ultimately, working together on a global scale provides significant knowledge that would be otherwise impossible to obtain.
